# Mobility Based Key Management Technique for Multicast Security in Mobile Ad Hoc Networks

**DOI:** 10.1155/2015/801632

**Published:** 2015-03-05

**Authors:** B. Madhusudhanan, S. Chitra, C. Rajan

**Affiliations:** ^1^Department of Computer Science, Er.Perumal Manimekalai College of Engineering, Hosur 635117, India; ^2^Er.Perumal Manimekalai College of Engineering, Hosur 635117, India; ^3^Department of Information Technology, KSR College of Technology, Tiruchengode 637211, India

## Abstract

In MANET multicasting, forward and backward secrecy result in increased packet drop rate owing to mobility. Frequent rekeying causes large message overhead which increases energy consumption and end-to-end delay. Particularly, the prevailing group key management techniques cause frequent mobility and disconnections. So there is a need to design a multicast key management technique to overcome these problems. In this paper, we propose the mobility based key management technique for multicast security in MANET. Initially, the nodes are categorized according to their stability index which is estimated based on the link availability and mobility. A multicast tree is constructed such that for every weak node, there is a strong parent node. A session key-based encryption technique is utilized to transmit a multicast data. The rekeying process is performed periodically by the initiator node. The rekeying interval is fixed depending on the node category so that this technique greatly minimizes the rekeying overhead. By simulation results, we show that our proposed approach reduces the packet drop rate and improves the data confidentiality.

## 1. Introduction

A set of wireless communication nodes performing self-configuration in a dynamic mode for formation of network excluding fixed infrastructure or centralized supervision is termed as mobile ad hoc network (MANET) [1]. It defines the set of wireless heterogeneous mobile nodes that performs communication with each other over multihop paths devoid of fixed infrastructure [2]. The key aim of MANETs is to extend the mobility criteria in autonomous, mobile, and wireless domain. The nodes in MANET perform as both hosts as well as routers for sending the packet to each other [3]. During ad hoc routing, every node in the network is permitted to discover its multihop path via the network to any other node [1]. The application of the MANET includes military battlefields, emergency search, and rescue locations, and so forth which requires quick deployment and active reconfiguration. Here the members make use of mobile devices for sharing the information [1].

The process of broadcasting the packets to a group of zero or more hosts recognized by a single destination address is termed as multicasting [1]. This implies that message is transmitted from one sender to several receivers or from multiple senders to multiple receivers. The merit of multicast technique is that it offers service to multiple users exclusive of network and resources overloading in the server [4]. The multicast technique is utilized by the application such as routing, neighbor discovery, key distribution and topology control. This technique is also used in identical data transmission from a single sender to several receivers that minimizes the network traffic and energy consumption [5].

The multicasting approach can enhance the efficiency of the wireless links for transmitting the multiple copies of messages in order to utilize the inbuilt broadcast nature of wireless transmission. Thus, multicast takes a major responsibility in MANET. The major aim of multicast routing protocol is to reduce the control overhead and processing overhead, enhancing the potentiality of multicast routing protocol, upholding the dynamic topology and avoids network loops and so on.

*Security in Multicasting in MANET*. The basic features of security in MANET are as follows: confidentiality guarantees that the network information cannot be revealed to the illegal unit. Integrity is essential to maintain the data to be transmitted among nodes without any change or degradation. Availability means that the services are demanded are available in timely manner without any potential issues in the system. The lack of authentication can cause the attacker masquerade any node and rules over the whole network. Nonrepudiation guarantees that the message forwarded cannot be refused by the message instigator [3].

*Key Management.* The methods of making, distributing, and updating the keys for a secure group communication application are termed as key management [6]. Encryption and reencryption are completed with the assistance of Traffic Encryption Keys (TEKs) and Key Encryption Keys (KEKs). In a secure multicast communication, each member possesses a key to encode and decrypt the multicast data. The method of updating and distributing the keys to the group members corresponds to rekeying operation. When each membership changes the rekey process is performed. However, throughout continual membership modulation, key management needs several exchanges per unit time for upholding forward and backward secrecies [7]. The secure multicasting is categorized into two types such as centralized and distributed scheme. The Group Controller (GC) performs group key management and only small loads are applied on the users of the group in case of centralized scheme. For distributed scheme, the key management is performed by each user to reinforce the load on the user [4].

## 2. Related Work

Chang and Kuo [8] have proposed a two-step secure authentication approach for multicast MANETs. A Markov chain trust model determines the Trust Value (TV) and the node with the highest TV is selected as CA server. The security analysis guarantees that this approach achieves a secure reliable authentication in multicast MANETs. Numerical results show that the analytical TV is very close to that of simulation under various situations. The speed of convergence of the analytical TV shows that the analyzed result is independent of initial values and trust classes. Huang and Medhi [9] have projected a secure group key management scheme for hierarchical mobile ad hoc networks to enhance each scalability and survivability of group key management for large-scale wireless ad hoc networks. A multilevel security model and a decentralized group key management infrastructure to come back through such a multi-level security model are projected. This approach reduces the key management overhead and improves resilience to any single point failure problem.

Bouassida and Bouali [10] have introduced an evaluation method for group key management protocols (GKMP). They have compared four main existing group key protocols, namely, scalable and efficient group rekeying protocol (GKMPAN) for ad hoc networks, Distributed Multicast Group Security Architecture (DMGSA), BALADE, and Hierarchical group key management protocol (Hi-GDH). In the above approaches, GKMPAN is an example for centralized approach. DMGSA approach belongs to distributed type key management scheme. BALADE protocol and Hi-GDH stand for decentralized approach. They have discussed the need for performance evaluation of GKMP's in the context of MANET's. Lin et al. [11] have proposed a new group key management protocol to reduce the communication and computation overhead of group key rekeying caused by membership changes. The protocol can handle synchronous and asynchronous rekeying operations, and a new *k*-node insertion algorithm is designed to further optimize the key tree in batch update operations. With strong encryption function and key derivation function, this protocol is provably secure. Simulation result shows that, compared to LKH, OFT, and ELK, SKD requires the least communication bandwidth and computation power, and it is efficient with binary key trees and asynchronous rekeying.

## 3. Proposed Work

The proposed technique uses Link Quality (LQ) and Reputation of nodes to identify them as strong or weak nodes. The multicast tree constructed with secure communication is based on the classified nodes and described in the subsections in detail.

### 3.1. Estimating Received Signal Strength

Here the proposed work makes use of the Friis free space propagation model to measure the received signal strength value. The received signal strength (RSS) is computed using the following formula [12]:

(1) $$\begin{array}{c} \text{RSS}=\alpha *\theta *S_{\text{tx}}, \end{array}$$

where *α* is a constant that relies on the wavelength and the antennas. *θ* is the channel gain. *S*_tx_ is the signal power of the transmitter.

RSS can be expressed in terms of the dB and dBm (dB milliWatts) as follows:

(2) $$\begin{array}{c} \text{RSS}\left[\text{dBm}\right]=10\log_{10}\alpha +\theta \left[\text{dB}\right]+S_{\text{tx}}\left[\text{dBm}\right]. \end{array}$$

#### 3.1.1. Link Quality

Link Quality (LQ) is estimated by ratio of the number of bits in error to the number of bits received (bit error rate) [13]:

(3) $$\begin{array}{c} \text{LQ}=\frac{b_{\text{rx}}}{b_{\text{error}}}. \end{array}$$

This value gets updated for every packet received at a node over a certain period. It depends on parameters such as the interference effect of the wireless channel, additive white Gaussian noise, and signal transmission range.

#### 3.1.2. Stability Index

Stability index (SI_*ij*_) is computed for a link to a neighbor based on the received signal strength, mobility, and link quality (using Sections 3.1.1, 3.1.2, and 3.1.3) [13]. SI_*ij*_ of a link between node *i* and node *j* is defined as follows:

(4) $$\begin{array}{c} {\text{SI}}_{ij}=\frac{\text{RSS}}{\text{LQ}}. \end{array}$$

#### 3.1.3. Estimation of Reputation of Nodes

Consider nodes *i* and *j*.

The recent satisfaction index (*P*_*ij*_) for node *i* about node *j* is computed as follows:

(5) $$\begin{array}{c} P_{ij}=f\left(i,j\right)-e\left(i,j\right), \end{array}$$

where *f*(*i*, *j*) is the percentage of packets originated from *i* that were forwarded by node *j* over the total number of packets offered to node *j*.

*e*(*i*, *j*) is the percentage of packets that were expired over the total number of packets offered to node *j*.

Thus, *P*_*ij*_ can be considered as the direct reputation of node *j*:

(6) $$\begin{array}{c} {\text{Rep}}_{ij}={\text{Rep}}_{ij\text{-pr}}*W_{\text{hist}}+P_{ij}*\left(1-W_{\text{hist}}\right), \end{array}$$

where Rep_*ij*-prev_ is the reputation value that node *i* had for node *j* before incorporating the most recent satisfaction index.

*W*_hist_ is a constant that reflects the level of confidence that node *i* has in the past observed reputation for its neighbor *j*.

The reputation index REP_*ij*_ is normalized using the following equation:

(7) $$\begin{array}{c} {\text{REP}}_{ij}=\frac{\text{RE}{\text{P}}_{ij}}{\text{ma}{\text{x}}_{t}\left({\text{REP}}_{ij}\right)}. \end{array}$$

max_*t*_ is the function that reports the maximum observation of REP_*ij*_ over time [14].

### 3.2. Classifying the Nodes

The nodes are categorized into two types, namely, strong and weak nodes. The steps involved in selecting the nodes are as follows.

1. Each node deployed in the network periodically exchanges a HELLO packet with its neighbor nodes.
2. By exchanging the hello packets, every node measures the RSS, link quality and mobility *M*_*j*_(*i*) of its neighbor nodes (explained in Sections 3.1.1 and 3.1.2).
3. Based on the measurement of RSS, link quality, and *M*_*j*_(*i*), each node computes the stability index (SI) of its neighbor nodes (explained in Section 3.1.3) and the values are stored in the neighbor table (NT).
4. The SI of each neighbor *N*_*i*_ is checked such that
5. Let SI_th_ be the predefined threshold value of Stability Index
6. If SI_*i*_ < SI_th_
7. Then The nodes are marked as weak nodes (*N*_*wi*_) and stored in NT
8. Else The nodes are marked as strong nodes (*N*_*si*_) and stored in NT
9. End if

**Figure 1:**
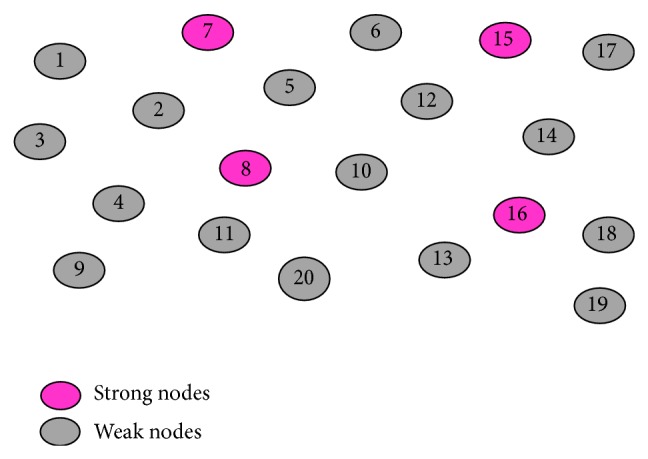
Selection of strong and weak nodes.

For example, consider the network in Figure 1. The nodes 7, 8, 15, and 16 are marked as strong nodes as their stability index is greater than the threshold value. Remaining nodes are marked as weak nodes as their stability index is less than the threshold value.

### 3.3. Multicast Tree Construction

The multicast tree construction phase involves two phases.

**Phase 1 .**

Each *N*_*wi*_ sends a child request message (CREQ) to each predetermined strong neighbor (*N*_*sj*_) stored in NT:

(8) $$\begin{array}{c} N_{wi}\overset{\text{CREQ}}{\to }N_{sj}. \end{array}$$

Upon receiving the CREQ message, *N*_*sj*_ sends a child reply message (CREP) to *N*_*wi*_:

(9) $$\begin{array}{c} N_{wi}\overset{\text{CREP}}{\leftarrow }N_{sj}. \end{array}$$

Every *N*_*wi*_ upon receiving CREP joins with *N*_*sj*_ as child nodes and respective *N*_*sj*_ becomes the parent node. Thus, for every weak node, there is at least a strong parent. *N*_*sj*_ then stores its child nodes information in a table.

**Figure 2:**
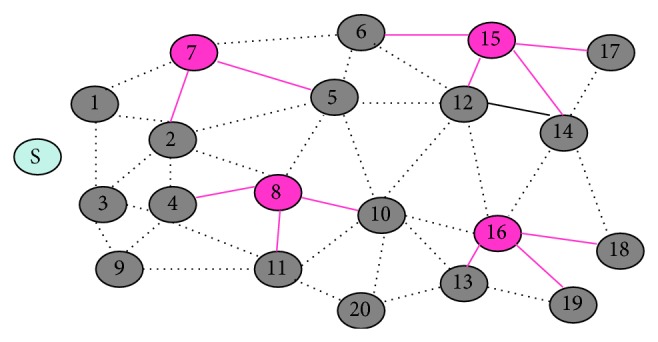
Phase 1: selection of child nodes.

For example, consider the network in Figure 2. The weak nodes 2 and 5 get attached with the strong node 7. Thus, nodes 2 and 5 become the child nodes for the strong parent node 7. In the similar manner, other strong nodes 8, 15, and 16 chooses their child nodes.

**Phase 2 .**

A multicast tree can be constructed and maintained using the periodic “JOIN_TREE” messages.

Each strong node *N*_*sj*_ periodically sends a “JOIN_TREE” message to the multicast source *S*:

(10) $$\begin{array}{c} N_{sj}\overset{\text{JOIN}\_\text{TREE}}{\to }S. \end{array}$$

*S* constructs a multicast tree consisting of the paths that “JOIN_TREE” pass through. There is only one path from the *S* to each *N*_*sj*_ of the multicast group.

**Figure 3:**
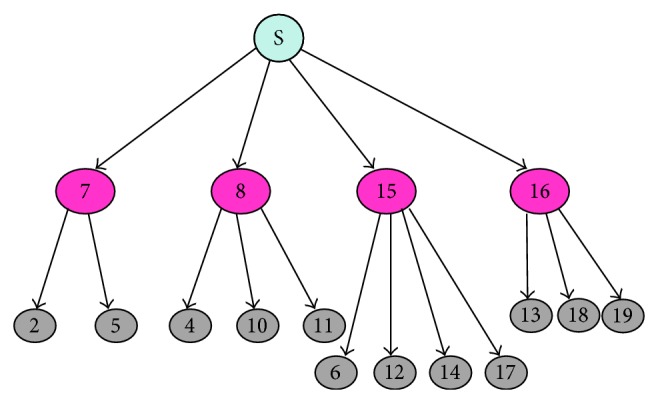
Multicast tree.

Figure 3 shows an example of a multicast tree constructed on a MANET. The parent nodes 7, 8, 15, and 16 sends JOIN_TREE message to *S*. *S* constructs a multicast tree consisting of the paths traversed by “JOIN_TREE” message.

#### 3.3.1. Secure Multicast Communication

When any node *N*_*i*_ wants to transmit multicast data to destination *D* in a secured manner, it performs the following steps.

**Figure 4:**
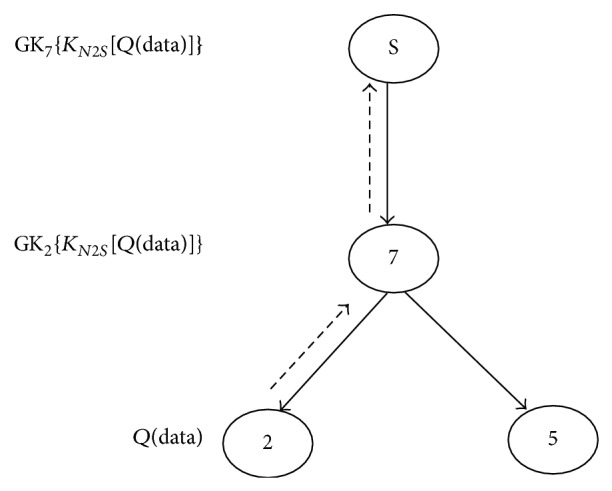
Secure data transmission.

1. Initially, *N*_*i*_ bounds the multicast data with hash message authentication code (*Q*) for ensuring the data integrity which is represented as *Q*(data).
2. *N*_*i*_ and *D* cooperatively compute the session key *K*_*iD*_ and *N*_*i*_ utilizes *K*_*iD*_ to encrypt *Q*[data]. This encrypted data is represented as *K*_*iD*_ [*Q*(data)]. Here, the session key is generated using Elliptic Curve Diffie-Hellman Key Management Agreement protocol (ECDH) [15].
3. Every member node holds a group key GK_*i*_. *N*_*i*_ again encrypts *K*_*iD*_ [*Q*(data)] with GK_*i*_ and it is represented as GK_*i*_{*K*_*iD*_[*Q*(data)]}. GK_*i*_ is the multicast group key, where, *i* = 1,2,…, *n*.
4. When any node along the path *N*_*i*_-*D* receives the GK_*i*_{*K*_*i*_[*Q*(data)]}, it decrypts the data using GK_*i*_ and encrypts it with GK_*i*_ again and forwards the encrypted data.
5. When *D* receives the encrypted data, it decrypts the data using its respective GK_*i*_ and session key *K*_*iD*_ and verifies the integrity of *Q*(data).
6. For example consider the network in Figure 4.

The node *N*_2_ wants to transmit the data packet to *S*. The data to be transmitted will be in the form: *Q*(data).

Initially, *N*_2_ and *S* cooperatively compute the session key *K*_*N*2*S*_ and *N*_2_ encrypts *Q*(data) with *K*_*N*2*S*_ which is represented as *K*_*N*2*S*_ [*Q*(data)]. *N*_2_ again encrypts *K*_*N*2*S*_ [*Q*(data)] with group key GK_2_ which is given as GK_2_{*K*_*N*2*S*_[*Q*(data)]}. This encrypted data is forwarded to *N*_7_.

*N*_7_ decrypts the data using the GK_2_ and encrypts again with GK_7_ and forwards it to *S* which will be in following form

(11) $$\begin{array}{c} \;\text{G}{\text{K}}_{7}\left\{K_{N2S}\left[Q\left(\text{data}\right)\right]\right\}. \end{array}$$

When *S* is receiving the encrypted data, it decrypts the information victimization GK_7_ and session key* K*_*N2S*_ and verifies the integrity of *Q*(data). If any changes happen throughout the transmissions, the receiving node detects the modifications in real time by validating the *Q*. The secured transmission of information between a node and therefore the supply is illustrated in Figure 4.

### 3.4. Detection of Attacker Nodes

When the data is not delivered at a reliable rate and optimum path quality, it is predicted that attack is detected. The attack detection technique depends on the capacity of *I* to detect the difference among the predicted PDR (PrP) and recognized PDR (ReP). The estimation of PrP and ReP is as follows.

PrP can be estimated from the Success Probability Product metric (SPP) at the concerned route.

SPP for a path of *n* links among *S* and *D* is given by

(12) $$\begin{array}{c} {\text{SPP}}_{S\to D}=\overset{i}{\underset{i=1}{\prod }}{\text{SPP}}_{i}, \end{array}$$

where the metric for each link *i* on the path is SPP_*i*_ = *Pr*⁡_succ_.

ReP of a route is determined by testing the continuity of the sequence number in received data packets. That is by dividing the number of received packets by the number of packets sent by the source over an interval of time.

ReP in terms of performance of packet delivery is given by the following equation:

(13) $$\begin{array}{c} \text{ReP}=\frac{P_{r}}{P_{s}}, \end{array}$$

where *P*_*r*_ is the average number of packets received by all receivers and *P*_*s*_ is the number of packets sent by the source.

Even if the attacker nodes drop all data packets, initiator nodes have the capacity to determine the ReP with the inclusion of the backup data packet authenticated by the source:

 If |PrP − ReP | >*η*
 Then
 The malicious behavior is detected by *I* since the particular route does not deliver the data at consistent level with optimal path quality.
 End if

#### 3.4.1. Isolation of Attacker Nodes

The steps involved in the isolation of attacker nodes are as follows.

**Step 1 .**

While detecting the malicious behavior, it temporarily recriminates the suspicious node by flooding a failure notice in the network that includes ID of recriminated and recriminator nodes and the period of recrimination.

**Step 2 .**

Until the recrimination is valid, metrics broadcasted by the recriminated node will not be taken into account and will be discarded during routing process.

**Step 3 .**

In case of transient network variations, the temporary recrimination scheme is taken into consideration.

**Step 4 .**

In temporary recrimination strategy, initially the time period of recrimination is computed in relative to the observed difference among PrP and ReP. This is performed with the intention that the recriminations caused by increase in metric values as well as malicious data dropping rate retains for longer duration than the recriminations caused by the transient network variations.

**Step 5 .**

In order to avoid the recrimination caused by attackers, a node is not permitted to announce a new recrimination prior to the expiry of the already announced recrimination.

**Step 6 .**

If the best metric is broadcasted by a recriminated node.

Then, the initiator node activates the recriminated node in addition to the best nonrecriminated node.

Step 6 reveals that the valid paths can still be utilized in spite of false recrimination of the strong nodes.

### 3.5. Rekeying Technique

Among the chosen *N*_*sj*_, some nodes have to be designated as initiators, which initiates the re- process. In this section, suppose that initiators are selected by centralized node considering reputation index (RI) of nodes. The initiators are selected based on the RI of nodes (explained in Section 3.1.3).

The direct reputation of node *N*_*sj*_ is given as

(14) $$\begin{array}{c} {\text{Rep}}_{ws}={\text{Rep}}_{ws\text{-pr}}*z+P_{ws}*\left(1-z\right), \end{array}$$

where Rep_*ws*-pr_ is the reputation value of *N*_*sj*_ contained in *N*_*wi*_ prior to the addition of recent satisfaction index. *z* is the constant that replicates the level of confidence possessed by *N*_*wi*_ for its *N*_*sj*_. *P*_*ws*_ is the recent satisfaction index for *N*_*wi*_ about *N*_*sj*_.

Thus, *N*_*sj*_ with high Rep_*ws*_ values are selected as initiators. The selected initiator starts the rekeying process periodically using the rekeying interval Rky_int_. Rky_int_ is the fixed parameter and rekeying procedure is demonstrated as follows.

 Let Rky_int_ be the initial time.
 Let Rky_max⁡_ represent the maximum thresholds for rekeying interval.
 Let Rky_min⁡_ indicate the minimum thresholds for rekeying interval.
 Let Rky_*t*_ represent the stop time.

**Figure 5:**
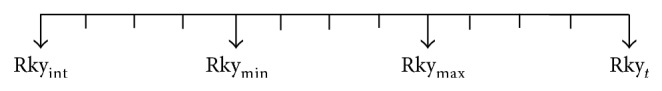
Rekeying time interval.

According to the rekeying interval, rekeying process is performed using the following cases.  Figure 5 shows the rekeying time interval.

**Case 1 .**

 If Rky_int_ > Rky_min⁡_
 then,
   the rekeying is performed for requested weak nodes from NT by the initiator.
 End if

**Case 2 .**

 If Rky_int_ > Rky_max⁡_
 then ,
   the rekeying is performed for requested strong nodes from NT by the initiator.
 End if

**Case 3 .**

 If Rky_int_ = Rky_*t*_
 
Then
   Rekeying is stopped and the timer is refreshed to start the new session.
 End if

The rekeying is performed in the weak node within minimum rekeying interval since they possess minimum stability index which causes them to frequently join or leave the network. In the strong nodes, rekeying is performed at the maximum rekeying interval since they have maximum stability index and their possibility to join or leave the network is less. This periodic rekeying reduces the repeated rekeying process that further reduces the overhead. In rekeying technique, the multicast group key (GK_*i*_) is rekeyed considering the three cases given above. The rekeying algorithm functions as follows [16].

According to the cases given above rekeying process is triggered. Initially, node *N*_*i*_ performs the ECDH key management agreement from leaf node to the source of multicast tree to obtain subgroup key cooperatively as

(15) $$\begin{array}{c} K_{N_{i}}+K_{N_{i+1}}+\cdots +K_{N_{n-1}}P. \end{array}$$

Here, *K*_*N*_*i*__ is the leaf node, *K*_*N*_*n*−1__ is the source, and *P* is the key generator in Diffe-Hellman. Finally, the generated subgroup chain reaches the source and it computes the new group key for the group. Once, the new group key is generated by the source, it unicasts it to the members securely.

Considering the tree structure given in Figure 4, node *N*_2_ and *N*_5_ are leaf nodes, *N*_7_ is the parent node of nodes 2 and 5, and *S* is the multicast source. Assume *N*_2_ invokes the rekeying process, and then the sequential process of rekeying is given below.

**Step 1 .**

*N*_2_ generates subgroup key as *K*_*N*_2__ + *K*_*N*_5__ *P* and transmits to *N*_7_.

**Step 2 .**

Node *N*_7_ computes the subgroup key as *K*_*N*_2__ + *K*_*N*_5__ + *K*_*N*_7__ *P* and forwards to the source.

**Step 3 .**

Finally, the source computes cooperative subgroup key as *K*_*N*_2__ + *K*_*N*_5__ + *K*_*N*_7__ + *K*_*S*_ *P* and then generates new group key as *K*_*i*_′ the source then unicasts the new group key securely to its member nodes.

## 4. Simulation Results

The proposed technique was simulated under different scenarios using varying number of receivers and varying the mobility of the nodes.

### 4.1. Simulation Model and Parameters

To analyze the performance of the proposed work NS2 [17] was used. In our simulation, the channel capacity of mobile hosts is set to the same value: 2 Mbps. We use the distributed coordination function (DCF) of IEEE 802.11 for wireless LANs as the MAC layer protocol. For multicasting, we used Multicast AODV (MAODV) [16] routing protocol. Simulations were carried out in 1500 meter × 1500 meter region for 50 seconds simulation time. We assume each node moves independently with the same average speed. All nodes have the same transmission range of 250 meters. In our simulation, the speed varied from 5 to 25 m/s and performance measured. The simulated traffic is Constant Bit Rate (CBR). In this simulation, we consider both the node capture and insider attacks. In node capture attack, a malicious attacker steals the credentials and secret keys from the legitimate nodes. An insider attacker is a malicious authenticated group member which may intimate false trust relations and injects false trust reporting. It may also inject packets *n* the network to disturb communications and consume the network resources. Our simulation settings and parameters are summarized in Table 1.

### 4.2. Performance Metrics

We compare our Mobility Based Key Management Technique (MBKM) with the traditional GKMPAN [10] and efficient clustering scheme for group key management (ECGK) [18]. We evaluate mainly the performance according to the following metrics.

*Average Packet Delivery Ratio.* It is the ratio of the number of packets received successfully and the total number of packets sent.

*Overhead.* It is the control overhead (in terms of packets) occurred in keying and rekeying operations.

*Packet Drop.* It is the average number of packets dropped at each receiver.

*Detection Accuracy.* It is the ratio of number of attacks detected to the number of attacks performed.

*Resilience.* It is the ratio of fraction of data compromised to the fraction of nodes compromised.

#### 4.2.1. Based on Receivers

In our first experiment, we vary the number of receivers per group as 10, 20, 30, 40, and 50 with speed 5 m/s.

*(i) Comparison with GKMPAN.* The proposed MBKM technique is compared with GKMPAN and the above performance metrics are evaluated by varying the group size.

**Figure 6:**
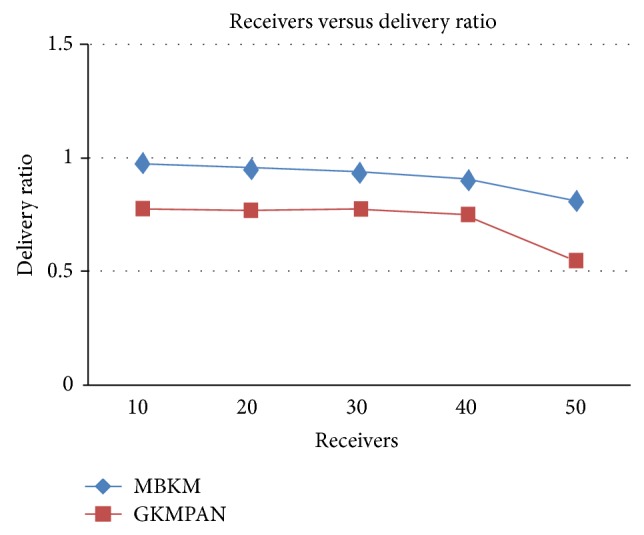
Comparison of delivery ratio with GKMPAN for varying receivers.

**Figure 7:**
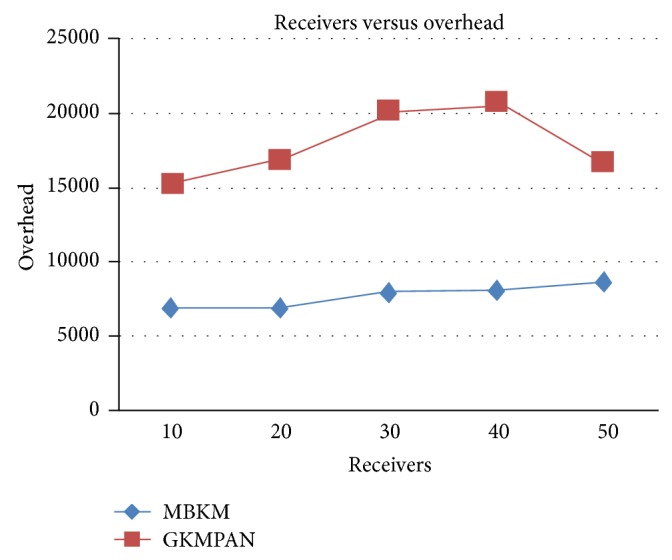
Comparison of overhead with GKMPAN for varying receivers.

**Figure 8:**
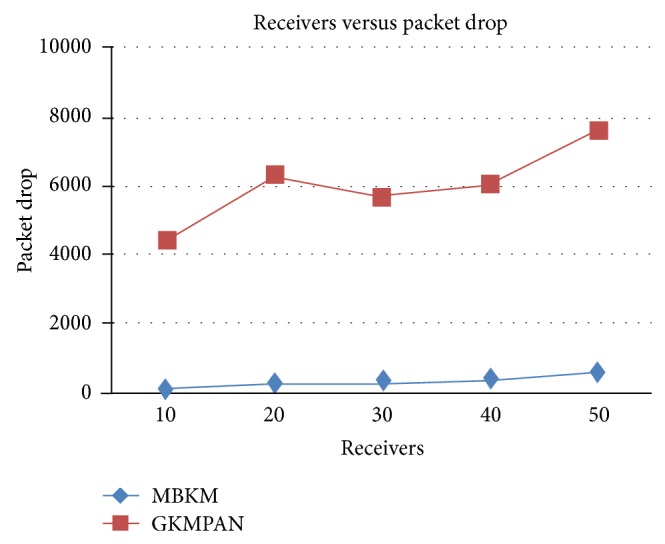
Comparison of packet drop with GKMPAN for varying receivers.

**Figure 9:**
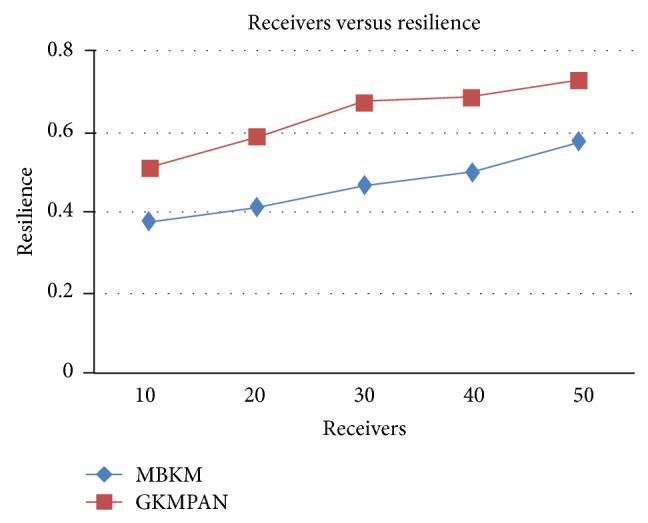
Comparison of resilience with GKMPAN for varying receivers.

Figures 6 and 8 present the packet delivery ratio and packet drop of both techniques, respectively, when the group size is increased from 10 to 50. From the figure, we can see that MBKM has 89% less packet drop than the existing GKMPAN techniques, since it assures high reliability using the strong nodes. Because of this reduced packet drop, the delivery ratio of the proposed MBKM is 23.57% higher than the GKMPAN technique.  Figure 7 presents the control overhead that occurred for both the techniques when the group size is increased. It can be seen that MBKM has 79.01% lesser overhead than the existing GKMPAN scheme, since it does not use the traditional multicast tree structure which involves large number of nodes.  Figure 9 presents the results for resilience for both the techniques when the group size is increased. It can be seen that MBKM has 30.96% lesser resilience than GKMPAN, since it has efficient rekeying technique.

**Figure 10:**
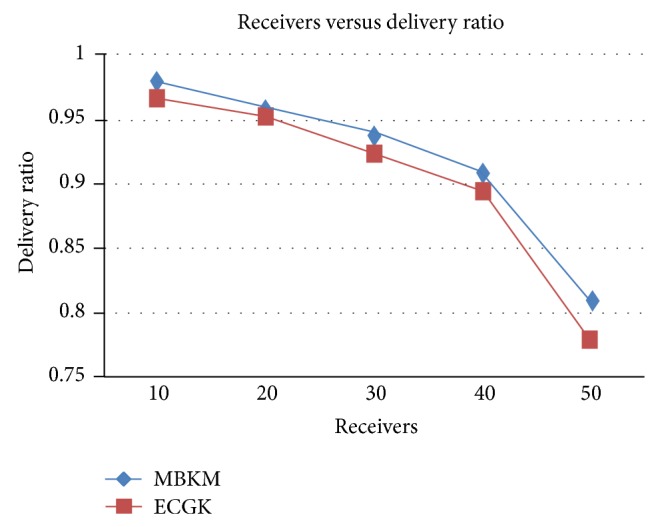
Comparison of delivery ratio with ECGK for varying receivers.

*(ii) Comparison with ECGK*. The proposed MBKM technique is compared with ECGK and the above performance metrics are evaluated by varying the group size. Figures 10 and 12 presents the packet delivery ratio and packet drop of both techniques, respectively, when the group size is increased from 10 to 50. From the figure, we can see that MBKM has 35.02% less packet drop than ECGK technique, since it assures high reliability using the strong nodes. Because of this reduced packet drop, the delivery ratio of the proposed MBKM is 1.82% higher than the ECGK technique.

**Figure 11:**
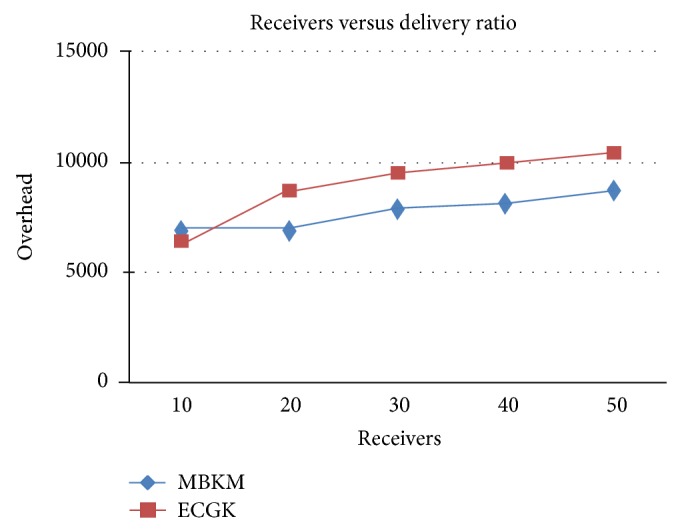
Comparison of overhead with ECGK for varying receivers.

**Figure 12:**
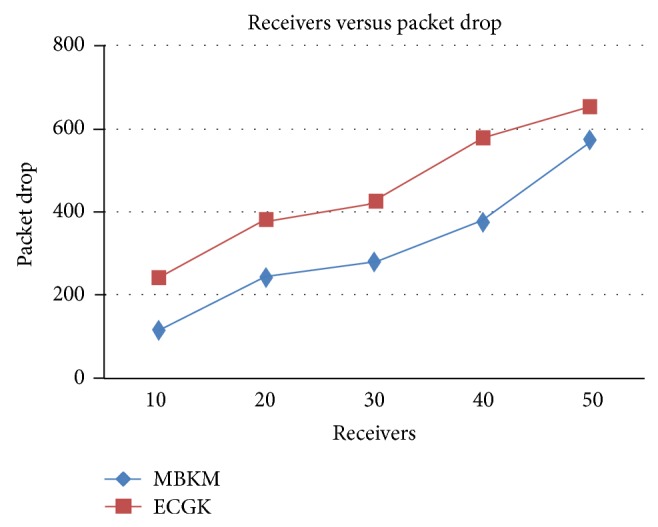
Comparison of packet drop with ECGK for varying receivers.

**Figure 13:**
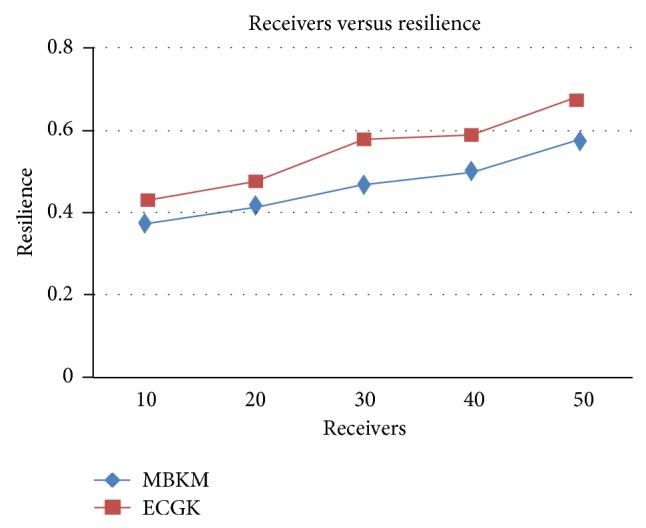
Comparison of resilience with ECGK for varying receivers.

Figure 11 shows the control overhead occurred for both the techniques when the group size is increased. It can be seen that MBKM has 15.32% lesser overhead than ECGK technique, since it does not use the traditional multicast tree structure which involves large number of nodes.  Figure 13 presents the results for resilience for both the techniques when the group size is increased. It can be seen that MBKM has 16.51% lesser resilience than GKMPAN, since it has efficient rekeying technique.

#### 4.2.2. Simulation Based on Node Speed

**Figure 14:**
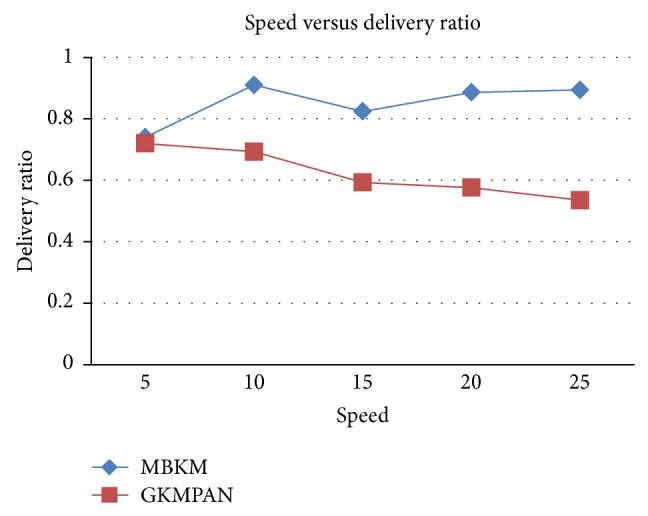
Speed versus delivery ratio.

**Figure 15:**
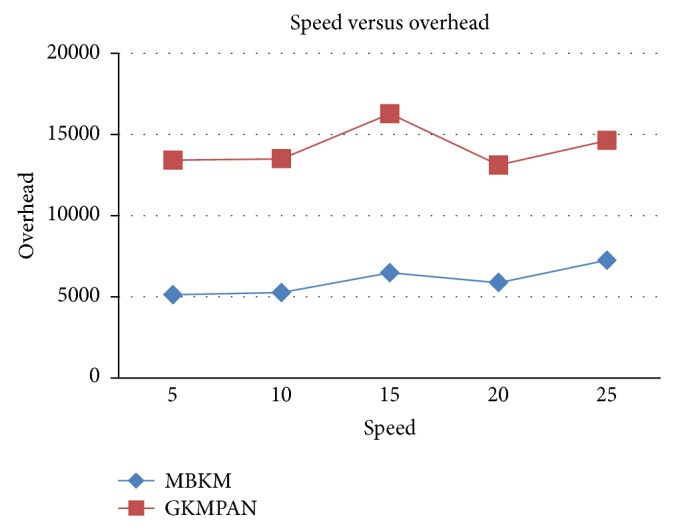
Speed versus overhead.

**Figure 16:**
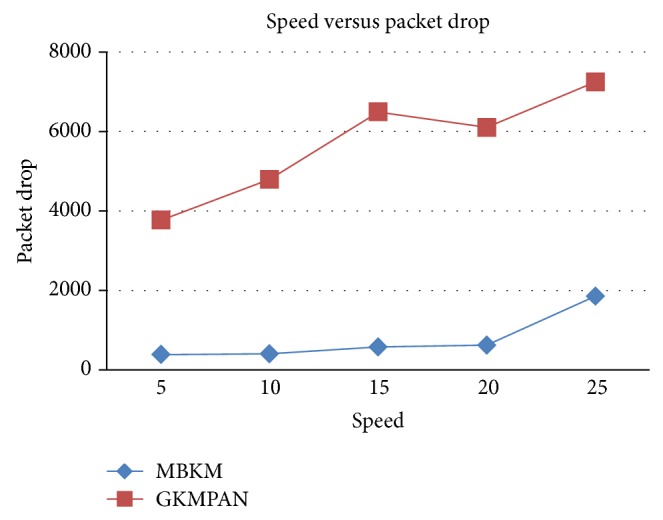
Speed versus drop.

In our second experiment we vary the speed of the mobile node as 5, 10, 15, 20, and 25 m/s for 10 receivers. Figures 14 and 16 present the packet delivery ratio and packet drop of both techniques, respectively, when the speed of the node is increased from 5 to 25 m/s. From Figure 11, we can see that the packet drop increases as the speed increases, due to disconnections and route breakages. But MBKM has 84% less packet drop than the existing GKMPAN techniques, since it uses stable and energy efficient nodes for routing. Because of this reduced packet drop, the delivery ratio of the proposed MBKM is 29% higher than the GKMPAN technique.  Figure 15 presents the control overhead occurred for both the techniques when the group is increased. It can be seen that MBKM has 56% lesser overhead than the existing GKMPAN scheme, since it does not use the traditional multicast tree structure which involves large number of nodes.

## 5. Conclusion

In this work, mobility based key management technique is used for multicast security in MANET. Initially the nodes are categorized into strong and weak nodes according to their stability index. The stability index is estimated based on the link availability and mobility. A multicast tree is constructed such that for every weak node, there is a strong parent node. When any node desires to transmit a multicast data to destination, a session key based encryption technique is utilized. The rekeying process is performed periodically by the initiator node which is chosen among the strong nodes based on the reputation index. The rekeying interval is fixed depending on the node category. For the weak nodes, the initiators perform rekeying within minimum rekeying interval as they possess minimum stability index. Whereas, for the strong nodes, the initiators perform rekeying at the maximum rekeying interval since their stability index is more and the possibility of their position change due to mobility is less. This technique minimizes the repeated rekeying process that further minimizes the overhead. By simulation results proposed approach reduces the packet drop rate and improves the data confidentiality.

## Figures and Tables

**Table 1:** Simulation parameters.

Number of receiver nodes	10, 20, 30, 40, 50
Area size	1500 × 1500
Mac	802.11
Radio range	250 m
Simulation time	50 sec
Traffic source	CBR
Rate	250 Kb
Mobility model	Random way point
Speed	5, 10, 15, 20 and 25.

## References

[1] Junhai L., Liu X., Danxia Y. (2008). Research on multicast routing protocols for mobile Ad-hoc networks. *Computer Networks*.

[2] Striki M., Baras J. S. Key distribution protocols for secure multicast communication survivable in MANETs.

[3] Rajan C., Shanthi N. S. (2013). Misbehaving attack mitigation technique for multicast security in mobile ad hoc networks (MANET). *Journal of Theoretical and Applied Information Technology*.

[4] Srinivasan R., Vaidehi V., Rajaraman R., Kanagaraj S., Chidambaram Kalimuthu R., Dharmaraj R. (2010). Secure group key management scheme for multicast networks. *International Journal of Network Security*.

[5] Lazos L., Poovendran R. (2007). Power proximity based key management for secure multicast in Ad hoc networks. *Wireless Networks*.

[6] Devi D. S., Padmavathi G. (2009). A reliable secure multicast key distribution scheme for mobile Adhoc networks. *World Academy of Science, Engineering and Technology*.

[7] Devaraju S., Padmavathi G. (2010). Dynamic clustering for QoS based secure multicast key distribution in mobile Ad hoc networks. *International Journal of Computer Science Issues*.

[8] Chang B.-J., Kuo S.-L. (2009). Markov chain trust model for trust-value analysis and key management in distributed multicast MANETs. *IEEE Transactions on Vehicular Technology*.

[9] Huang D., Medhi D. (2008). A secure group key management scheme for hierarchical mobile Ad hoc networks. *Ad Hoc Networks*.

[10] Bouassida M. S., Bouali M. On the performance of group key management protocols in MANETs.

[11] Lin J.-C., Huang K.-H., Lai F., Lee H.-C. (2009). Secure and efficient group key management with shared key derivation. *Computer Standards & Interfaces*.

[12] Sridhara V., Bohacek S. (2007). Realistic propagation simulation of urban mesh networks. *Computer Networks*.

[13] Biradar R., Manvi S., Reddy M. (2010). Mesh based multicast routing in MANET: stable link based approach. *International Journal of Computer and Electrical Engineering*.

[14] Zakhary S. R., Radenkovic M. Reputation-based security protocol for MANETs in highly mobile disconnection-prone environments.

[15] (2012). *Elliptic Curve Cryptography, Version 2.0, Technical Guideline*.

[16] Lin H.-Y., Chiang T.-C. (2011). Efficient key agreements in dynamic multicast height balanced tree for secure multicast communications in Ad Hoc networks. *EURASIP Journal on Wireless Communications and Networking*.

[17] http://www.isi.edu/nsnam/ns/.

[18] Drira K., Seba H., Kheddouci H. (2010). ECGK: an efficient clustering scheme for group key management in MANETs. *Computer Communications*.

